# Hallmarks of nursing students exhibiting unsafe clinical practices: a qualitative study

**DOI:** 10.1186/s12912-025-03093-x

**Published:** 2025-04-18

**Authors:** Akram Ghahramanian, Mostafa Ghasempour, Vahid Zamanzadeh, Leila Valizadeh, Laura A. Killam, Majid Purabdollah, Mohammad Asghari-Jafarabadi

**Affiliations:** 1 Medical Education Research Center, Health Management and Safety Promotion Research Institute, Tabriz University of Medical Sciences, Tabriz, Iran; 2 Department of Medical-Surgical Nursing, School of Nursing and Midwifery, Tabriz University of Medical Sciences, Tabriz, Iran; 3 Department of Pediatric Nursing, School of Nursing and Midwifery, Shahid Beheshti University of Medical Sciences, Tehran, Iran; 4 School of Nursing, Cambrian College, Sudbury, ON Canada; 5 School of Nursing, Faculty of Education and Professional Studies, Nipissing University, North Bay, ON Canada; 6 Department of Nursing, Faculty of Nursing, Khoy University of Medical Sciences, Khoy, Iran; 7 School of Public Health and Prevention Medicine, Faculty of Medicine, Nursing and Health Sciences, Monash University, Melbourne, VIC 3800 Australia; 8 Department of Psychiatry, School of Clinical Sciences, Monash University, Clayton, VIC 3168 Australia

**Keywords:** Nursing students, Unsafe practice, Patient safety, Qualitative research

## Abstract

**Background:**

Maintaining and promoting patient safety is a shared responsibility among all participants in the health care system. Educators are required to balance patients’ rights to receive safe care and create a suitable and safe environment for nursing students to learn. Therefore, early identification of students with unsafe clinical practice and intervention may be important measures for improving patient safety. Therefore, the present study was conducted with the aim of identifying the main hallmarks of nursing students with unsafe clinical practice.

**Methods:**

This qualitative study was conducted with 19 faculty members, nursing students, and supervisors of medical centers. Data collection was performed through purposive sampling and semi structured interviews. Data analysis was performed via conventional qualitative content analysis via MAXQDA10 software.

**Results:**

The results of the study led to the identification of 2 main categories, “Underdeveloped knowledge and cognitive capacity” and “Underdeveloped personal-professional capacity”, and 6 and 4 subcategories, respectively, as the main hallmarks for identifying students with unsafe clinical practice.

**Conclusion:**

The findings of this qualitative study expand our understanding of the hallmarks of nursing students with unsafe clinical practice. Undergraduate nursing students with unsafe clinical practice may not have acquired sufficient development and progress in terms of knowledge, skills, and personal-professional characteristics or may not be able to demonstrate them in their practices. Nursing schools must ensure that students have the necessary knowledge, skills, competencies, and personal-professional characteristics to participate in clinical training programs. It is recommended that students with unsafe clinical practices be identified early so that patient safety is maintained and that students are supported in order to correct their weaknesses and improve.

**Supplementary Information:**

The online version contains supplementary material available at 10.1186/s12912-025-03093-x.

## Introduction

Clinical education is one of the most important parts of nursing education programs. Students have the opportunity to develop their knowledge and skills, practice, and become competent nurses through direct contact with patients in different healthcare settings [1]. Clinical educators play a key role in shaping students’ clinical experiences. These clinical educators need to engage in self-preparation and planning for learning opportunities in the clinical setting, facilitate student learning, evaluate students on an ongoing basis, role model professional practice, and assess nursing student competence [2]. Despite these important roles, Leighton et al.‘s (2021) review revealed serious concerns about the lack of evidence supporting how nurse educators assess and impact learning in traditional clinical environments [3]. Clinical educators must aim to balance patients’ rights to receive safe care while creating an appropriate and safe environment for independent student learning [2]. Promoting student learning while maintaining patient safety means using appropriate strategies for the early identification of students who exhibit unsafe clinical practices [4]. Moreover, Gcawu and van Rooyen (2022) conducted an integrative review and reported that, of the studies that refer to work-based assessment in clinical education, none introduce tools for evaluating the safety of students’ clinical practice [5].

Researchers have shown that clinical educators find it challenging to identify, evaluate, and manage the unsafe patterns and practices of students [4, 6]. Several factors, such as an unclear definition of behavioral characteristics that indicate unsafe practice [1, 4, 7], instructors’ contradictory interpretations of clinical behaviors that are unsafe [1, 8], social pressures [9, 10], the absence of clear frameworks [1, 10], fear of reprisal [10–12], uncertainty in their decision to fail students who act in unsafe ways [1, 12–15] and a lack of support [9], are contributing factors to this challenge.

Although the concept of unsafe clinical practice has been emphasized as a phenomenon that needs intervention by several researchers [10, 16], a lack of consensus about how to define and intervene when students exhibit unsafe practices remains in the literature [17]. Unsafe clinical practices can include many actions or behaviors, such as a lack of knowledge, skill, or clinical judgment or any unprofessional or unethical behavior of the student, in such a way that the physical, emotional, psychological, or environmental security of the client, themselves or other health care personnel and peers are exposed to danger or harm [18]. Killam et al. (2013) reported that for first-year students, practicing safely during clinical practice is complex and involves multiple dimensions of personal, professional, and programmatic factors [19]. Furthermore, students may work to conceal indicators that their practice may be unsafe to pass the placement [20]. As such, clinical educators need to be aware of signs that may signal that a student could be practicing in unsafe ways; we refer to these signals as hallmarks of unsafe practice.

An initial understanding of the hallmarks of students exhibiting unsafe clinical practices has been identified in previous studies. For example, Luhanga et al. (2008) classified the behaviors associated with unsafe student performance into four subcategories: inability to demonstrate knowledge and skills, attitude problems, unprofessional behavior, and poor communication skills [21]. Killam et al. (2011) conducted an integrative review and identified three main themes signaling unsafe student practice: ineffective interpersonal interactions (including poor communication and relational difficulties), inadequate knowledge and skills (including limited cognitive capacity and other weak skills), and an unprofessional image (such as inappropriate attitudes and behaviors, poor accountability and dishonesty) [4]. The characteristics identified in other studies include irregularity and delay in attendance [7, 22], lack of responsibility [1, 15], anxiety [22], inappropriate behavior with patients, not asking questions when needed [7], not having enough enthusiasm or motivation to learn and perform care [22, 23], ignoring learning opportunities, showing unethical or unprofessional behavior [9], attitude problems [23], overconfidence [18, 23], low self-confidence [4] and difficulty in accepting constructive feedback [23]. Further exploration of the hallmarks of students with unsafe clinical practices is needed to deepen our understanding of which characteristics may be of immediate concern for clinical educators.

Patient safety should be the highest priority in the fields of clinical care and education, and clinical educators are in an important and critical position to ensure that nursing students maintain high standards of safe clinical practice. Unfortunately, there is not enough evidence to guide educators in difficult situations to identify nursing students with unsafe clinical practice [1, 7]. Clinical educators and preceptors are left with the important responsibility of judging whether the student is safe or unsafe and deciding on corrective actions [23]. A lack of support in the form of clear policies, procedures, and definitions of unsafe practice that involve institutional support are among the contributing factors to the problematic phenomenon of failure to fail nursing students who are not able to practice safely [13, 24]. Conducting more studies to identify these hallmarks may help clinical educators and institutions develop a consensus on what student practices are unsafe. The perspectives of persons directly involved in clinical education are also essential for informing policy decisions.

The concept of ‘hallmarks’ emphasizes the key features or patterns that serve as red flags for potential risks or deficiencies in patient care. These hallmarks provide important insights for identifying and addressing areas of concern that may compromise patient safety and the quality of nursing practice [21, 25]. This usage of ‘hallmarks’ aligns with the qualitative nature of our study, which aims to explore and understand the underlying factors and behaviors contributing to unsafe clinical practice among nursing students. Identifying these hallmarks is crucial for the development of targeted interventions and educational strategies to improve the competence and professionalism of future nurses. Although previous research has investigated unsafe clinical practices among nursing students, the focus has often been on general safety concerns rather than on systematically defining and categorizing unsafe behaviors. Additionally, most existing studies have used quantitative approaches, which, due to their structured nature, may not fully capture the complexity and depth of this phenomenon. This study is among the few that explore this concept qualitatively, allowing for a deeper understanding of the meanings and experiences associated with unsafe clinical practices. There remains a gap in developing a structured framework that enables early identification and intervention for students who demonstrate unsafe clinical performance. Our study fills this gap by employing a rigorous qualitative approach, gathering data from faculty, clinical educators, and students to develop a comprehensive taxonomy of unsafe practice hallmarks. By providing a comprehensive categorization of unsafe practice hallmarks, this research can equip educators with the tools needed for early identification and intervention, leading to the development of targeted educational strategies and, ultimately, enhanced patient safety and nursing education. Therefore, the present study was conducted with the aim of identifying the main hallmarks of nursing students with unsafe clinical practice.

## Methods

### Study design

The aim of this study was to discover the hallmarks of nursing students with unsafe clinical practice from the perspective of faculty members, clinical educators, preceptors, and nursing students via an inductive qualitative research method. Qualitative research is a systematic and subjective approach that enhances insight, understanding, and awareness of human experiences. Inductive methods, as a core component of qualitative analysis, provide a comprehensive framework for examining complex phenomena. This process involves deep engagement with the data through meticulous reading and interpretation, allowing researchers to identify patterns and uncover underlying meanings [26, 27]. Such insights are essential for informing policy development, particularly in areas such as managing potentially unsafe student behaviors, where a nuanced understanding of contextual factors is crucial. This approach also helps unearth what drives human behaviors and decisions. This knowledge may help inform clinical education problem-solving and the development of interventions and policies in nursing education programs to improve competencies for students and treatment outcomes for patients [28]. Therefore, to discover and explain the dimensions of the phenomenon in question and reveal a deep understanding of the social world of participants, an inductive qualitative research approach was used [26].

### Participant recruitment

A purposive sampling strategy was employed to ensure diverse perspectives from different stakeholders in nursing education. Participants included 3 faculty members from an academic setting, 4 clinical educators supervising student placements, 6 nursing students, 3 preceptors, 1 head nurse, 1 educational supervisor, and 1 patient safety supervisor, each of whom provided unique insights into unsafe clinical practices. This diversity was intended to ensure comprehensive data collection by integrating expert evaluations from faculty members and clinical educators alongside first-hand experiences from nursing students and preceptors. The variety of roles contributes to a holistic understanding of the hallmarks of unsafe clinical practices.

The study was conducted in Tabriz, Iran, at a School of Nursing affiliated with government-funded teaching hospitals. These specialized facilities offer nursing students clinical experiences across various departments, including internal medicine, surgery, and critical care. Hospitals are well equipped to train nursing students in real-world healthcare settings, ensuring exposure to a wide range of patient care situations. The inclusion criteria prioritized clinical educators and managers with extensive experience in instructing and collaborating with nursing students’ education. Participant selection included nursing students enrolled in at least their 7th semester to ensure adequate clinical exposure. In Iran, the undergraduate nursing program consists of eight semesters (four years), with the 7th and 8th semesters focused primarily on intensive clinical training.

Initially, participants were simultaneously selected from clinical instructors and students; as the study progressed, purposive sampling facilitated the inclusion of participants with diverse perspectives, aiming for maximum variation in age, gender, educational level, hospital type, and department-specific experience. Continuous comparison and data analysis, along with memo writing during data collection, guided the researcher’s intentional selection of subsequent participants. Sampling proceeded until no additional new information, categories, or themes emerged. Data saturation was confirmed through a thorough re-examination of codes and categories by three research team members, with input from two external experts. Two additional interviews were conducted to further validate the data saturation.

### Researcher characteristics and reflexivity

The authors of this article are faculty members who are primarily involved in clinical education for undergraduate nursing students. Through their roles as clinical instructors, they have frequently observed situations where patient safety was at risk during student training in clinical environments, underscoring their commitment to upholding patient safety. This experience spurred their interest in conducting a study to examine the traits of students exhibiting unsafe clinical practices. Drawing on the insights of students, clinical instructors, and those involved in nursing education, their goal was to facilitate early identification of students with unsafe practices, address these issues promptly, and ultimately protect patient safety. Previous studies have highlighted concerns surrounding students with unsafe clinical behaviors [4, 7, 20, 21]. Prior to initiating this research, qualitative researchers must recognize and set aside personal thoughts, perspectives, and assumptions related to the subject through the bracketing process, thus minimizing potential biases in data collection and analysis [29]. Given that the researchers had prior professional relationships with some participants, steps were taken to minimize potential bias in data collection and analysis. Reflexivity was maintained through bracketing. Bracketing techniques were employed throughout the data collection process. Specifically, before each interview, researchers engaged in a bracketing exercise, writing down their pre-conceived notions about unsafe clinical practices and the participants’ experiences. This helped to consciously set aside these preconceptions during the interviews and reduce their influence on questioning and interpretation. Additionally, reflexive journaling was used throughout the study to document and mitigate researcher influence. Researchers regularly recorded their thoughts, feelings, and reactions to the data, allowing them to track potential biases and adjust their approach accordingly. To further enhance credibility, member checking was conducted after initial analysis, where participants reviewed their transcripts and the preliminary findings to ensure accuracy and prevent bias in interpretation. Participants were encouraged to provide feedback on the researchers’ interpretations, and any disagreements were carefully considered and addressed. Finally, data analysis was carried out collaboratively by multiple researchers, ensuring a balanced interpretation of findings and reducing the influence of individual perspectives. Content analysis was conducted independently by at least two researchers, and discrepancies were discussed until consensus was reached. This helped to ensure that interpretations were grounded in the data rather than being driven by individual bias.

### Data collection

Data collection was conducted between December 2021 and September 2022 via in depth, face‒to-face semistructured interviews that typically lasted from 40 to 60 min (average duration = 50 min). The participants selected the time and location for each interview, which were held in private spaces within hospitals and university offices to ensure comfort and facilitate open discussions. All the interviews were conducted by the second author, with the first author observing the initial three sessions to ensure consistency. Each interview was audio recorded with participants’ consent to maintain data accuracy, and field notes were taken during and after each session to capture contextual details and nonverbal cues. Initial warm-up questions were posed, followed by a general inquiry. The interview guide utilized in this study was developed as part of a comprehensive doctoral research project aimed at exploring safe and unsafe practices in clinical education, capturing diverse perspectives across multiple datasets. While the core guide remained the same, the questions were specifically tailored to each participant group. Faculty members and clinical educators were asked about their experiences in identifying and managing unsafe practices, whereas nursing students discussed their own experiences with safe and unsafe clinical practice. While not all participants themselves directly exhibited unsafe practices, all participants were able to identify, describe, and reflect on unsafe behaviors they had observed in clinical settings. The classification of a behavior as “unsafe practice” was based on participants’ descriptions of actions that posed a potential or actual risk of harm to patient safety, such as medication errors, inadequate infection control practices, or failure to follow established protocols. As clarified in the Data Collection and Analysis section, these criteria for “unsafe practice” were not predetermined but rather emerged inductively from the thematic analysis of the interview data, ensuring that the classification was grounded in the participants’ lived experiences and perceptions.

The key questions posed by the second author included “What are your experiences with safe and unsafe practices, and what are your feelings regarding your own unsafe practices? In your view, what characteristics define a safe or unsafe student?”

Educators were asked to highlight specific behaviors they identified as unsafe among students in clinical settings and internships and to reflect on their roles as evaluators responsible for upholding patient safety. Nursing students, in turn, discussed their experiences with unsafe practices, both their own practices and those observed in others. To further explore participants’ responses, follow-up questions such as “What do you mean by situational awareness? Can you provide an example? What was your experience?” were used to probe, direct, and verify their statements. The participants were encouraged to elaborate on and clarify the concepts they introduced during the discussions. To enhance data comprehensiveness, the interview guide was refined through continuous comparison and analysis during data collection. The updated supplementary materials now include a detailed breakdown of the interview questions for each participant group (see the Supplementary File).

### Ethical considerations

This study was conducted as a part of a doctoral dissertation in nursing after receiving the ethical approval code from the Regional Research Ethics Committee of Tabriz University of Medical Sciences (Ethical Code: IR.TBZMED.REC.1400.608). All participants were fully informed about the study’s aims, procedures, confidentiality of their information, and their right to withdraw at any time without any consequences. Written informed consent was obtained from each participant before the interviews were initiated, ensuring their voluntary and informed participation.

### Data analyses

A conventional content analysis approach was employed, allowing categories for analysis to emerge directly from the data itself rather than being predetermined. This method systematically classifies collected data to reveal both explicit and implicit themes and patterns, thereby generating knowledge and offering fresh insights. Each session of audio recordings was transcribed verbatim, capturing all verbal and nonverbal cues, including silences and emphasis [30].

The transcriptions were then reviewed multiple times by the first and second authors (MG and AG) to foster deep engagement with the data. The text was segmented into semantic units at varying levels, including words, sentences, and paragraphs. Under the guidance of the research team (MA, AG, VZ, and LV), the first author systematically coded the interviews. Initial categories were developed through ongoing comparisons of similarities and differences in the codes generated. The stability of the coding was subsequently assessed via the Holsti method to verify the level of agreement between the primary researcher and an external coder not affiliated with the research team [31]. A Holsti coefficient exceeding 0.7 was deemed acceptable [32].

Following the establishment of adequate coding consistency, coding rules were systematically applied across all sections of the text, and the coding process continued until no new codes, categories, or themes were identified. Regular meetings of the research group were conducted throughout the coding phase to review the content of the codes, verify coding stability, and organize categories. The codes, categories, and subcategories were ultimately developed through processes of comparison, evaluation, feedback, and ongoing interpretation. Data management during the analysis was facilitated via MAXQDA software, version 2010 (VERBI GmbH, Berlin, Germany). Table 1 illustrates the progression of the analysis from raw data to the core concepts. Authors MG, AG, VZ, and LV were actively involved in both the analysis and the preparation of this section.

### Trustworthiness of the data

To ensure the trustworthiness of the data, the criteria established by Lincoln and Guba, including credibility, dependability, transferability, and confirmability, were employed [33]. The researcher fostered a long-term relationship with participants and utilized member checking, aimed for maximum diversity among participants, conducted external checks, engaged in peer reviews, and practiced bracketing to increase the credibility of the findings. Furthermore, the audibility of the analysis was validated by two experts (MA and LK), who reviewed the analytical process.

**Table 1:** The sample of data analysis for the emerging subcategory of nontechnical skills and capabilities

Category	Subcategory	Main concepts	Sample codes	Sample supporting quote
Underdeveloped knowledge and cognitive capacity	Insufficient nontechnical skills and	1. Failure to manage priority tasks	Failure to identify and prioritize patient problems Inability to prioritize tasks	Most of us students cannot manage and prioritize our work well. This makes us forget some things, or do it half and half and patient care will be missed. (Participant 4, Academic member)
		2. Lack of situational awareness	Inability to identify a patient’s poor condition Inability to identify and take appropriate action according to the critical condition of the patient	A student who performs unsafely cannot recognize the bad condition of the patient and is just doing the procedures. The patient’s saturation was 82% and he had severe respiratory distress. Instead of him telling us the condition of the patient, I warn him that if you do not see that your patient is not breathing well, then why do not you inform him and start oxygen for him? He says very relaxed, I did not understand. (Participant 7, Clinical educator)
		3. Defects/delays in clinical decision-making	Defects in clinical decision making Inability to put together patient data and make clinical decisions based on it	Most of us have problems in clinical decision-making. We cannot diagnose the serious condition of the patient; we have done investigations and collected the data; we cannot put the pieces of data together and choose the best thing we can do for the patient. (Participant 10, Nursing Student)
		4. Deficits in teamwork and interprofessional cooperation	Inability to cooperate with the doctor to perform procedures. Unwillingness to work as a team with doctors and medical students.	The student is sitting at the nursing station and I say to him, why are not you with your patient?! He says that the interns are there and they are taking a history and they are going to put a Foley catheter on him, I say go and be with them and learn how to work and cooperate with them. Discuss the patient’s issues with them and exchange information. (Participant 6, Clinical educator)
		5. Deficits in interpersonal communication skills	Poor communication with the patient Poor communication with the care team.	According to my many years of experience in clinical settings and working with different students, I say that people with defects in interprofessional communication and even patient communication are unsafe people. They are more likely to make mistakes in their work. (Participant 5, Academic member)
		6. Failure to report information and important matters to others	Hiding an error occurred Failure to report errors to others Delay in receiving important interventions due to failure to report errors	Failure to report important things such as the mistakes they make is one of the characteristics of an unsafe student. Students who hide and do not report the error will delay intervention to prevent further complications. (Participant 17, Head nurse)

## Results

The results of this study are part of a Ph.D. dissertation in nursing, which was conducted to identify the characteristics of students with unsafe clinical practices [34]. Table 2 shows some of the demographic characteristics of the participants in the study. In the present study, the categories “Underdeveloped knowledge and cognitive capacity” and “Underdeveloped personal-professional capacity” were extracted from the data analysis as the hallmarks identifying students with unsafe clinical practices. Table 3 shows the categories, subcategories and attributes that indicate the unsafe clinical practices of nursing students (Table 3). The other main finding, “self-presentation,” has been reported elsewhere [20].

**Table 2:** Some sociodemographic characteristics of study participants

Participant ID	Age	Sex	Education level/semester	Employment status	Clinical background (years)	Educational background (years)
1	40	Female	Ph.D. ^a^	Clinical educator	15	8
2	42	Male	Ph.D.	Clinical educator	17	10
3	35	Male	M.Sc. ^b^	Academic member	8	6
4	49	Female	Ph.D.	Academic member	13	15
5	53	Female	Ph.D.	Academic member	20	15
6	38	Male	Ph.D.	Clinical educator	11	8
7	54	Female	M.Sc.	Clinical educator	20	18
8	24	Female	Nursing student/8th	Nursing Internship	-	-
9	30	Male	Nursing student/7th	Nursing Internship	-	-
10	24	Female	Nursing student/7th	Nursing Internship	-	-
11	23	Male	Nursing student/6th	Nursing Internship	-	-
12	34	Male	Nursing student/8th	Nursing Internship	9 (LPN)^c^	-
13	23	Female	Nursing student/7th	Nursing Internship	-	-
14	30	Male	M.Sc.	Preceptor/nurse	7	2
15	37	Female	M.Sc.	Preceptor/nurse	12	3
16	35	Male	M.Sc.	Preceptor/nurse	10	3
17	46	Female	BSN ^d^	Head nurse	21	-
18	47	Male	M.Sc.	Educational supervisor	22	10
19	35	Female	BSN ^**d**^	Safety supervisor	10	-

^**a**^ Doctor of Philosophy, ^**b**^ Master of Science in Nursing, ^**c**^ Licensed practical nurse, ^**d**^ Bachelor of Science in Nursing

**Table 3:** Illustration of the categories, subcategories, and attributes indicating hallmarks of unsafe practices

Categories	Subcategories	Attributes
I. Underdeveloped knowledge and cognitive capacity	1. Lack of Technical skills and abilities	1. failure in initial and continuous assessment
		2. Failure to implement/demonstrate clinical skills.
		3. Failure to properly administer medication
	2. Insufficient nontechnical skills and abilities	1. Failure to manage priority tasks
		2. Lack of situational awareness
		3. Defects/delays in clinical decision-making
		4. Deficits in teamwork and interprofessional cooperation
		5. Deficits in interpersonal communication skills
		6. Failure to report information and important matters to others
	3. Limited knowledge	1. Deficits in knowledge about theoretical topics, diseases, care, and working with equipment
		2. Deficits in Knowledge of safety principles, precautions, and preventive measures
		3. Deficits in knowledge of duties and actions accordingly
	4. Unrefined clinical reasoning and judgment	1. Deficits in intellectual movement from data to nursing diagnosis
		2. Failure to rely on learnings and scientific evidence in practice
		3. Failure to choose appropriate solutions/nursing activities for the patient’s problems
	5. non-Patient-centered care	1. Failure to provide holistic care for the patient
		2. Focusing only on performing the procedure for the patient instead of being patient-centered
		3. Failure to consider the preferences and opinions of the patient/family caregivers
	6. Inappropriate attitudes about nursing	1. Procedure-oriented and nonacademic view of nursing
		2. Unenthusiastic attitudes to nursing
		3. Self-centeredness/doing procedures arbitrarily and without obtaining permission
III. Underdeveloped personal-professional capacity	1. Lack of conscientiousness	1. Lack of knowledge/noncompliance with rules and regulations
		2. Lack of moral sensitivity/deficiency in ethics and professional integrity
		3. Lack of responsibility and professional accountability
		4. Lying (dishonesty) and data fabrication
		5. Ignoring learning opportunities
		6. Irregularity and ineffective attendance at an internship
	2. Deficit in psychological readiness	1. Unmanaged anxiety, stress, and fear
		2. Difficulty in adapting to the clinical environment
		3. Feeling of inadequacy and low self-esteem
		4. Student dependence on others (dependent personality)
	3. Inappropriate professional appearance	1. Failure to comply with the uniform set determined by the institution
		2. Improper hygiene and grooming
	4. Inappropriate behaviors	1. Uncontrolled anger and aggression
		2. Fatigue and sleepiness
		3. Unprofessional behaviors in clinical settings.

## Category 1: Underdeveloped knowledge and cognitive capacity

The participants reported that safe clinical practice means that students must have sufficient confidence in their ability to perform clinical skills and have the necessary technical and nontechnical preparation to be present in clinical environments. Additionally, in addition to having professional attitudes, students should have sufficient preparation in terms of knowledge and awareness to perform and establish safe practices. They should use clinical reasoning and judgment combined with a patient-centered view to provide safe care and practices. When students are unable to demonstrate any of these characteristics of safe practice, it is considered a hallmark of unsafe practice. Below are the defining subcategories of this category of unsafe practice with quotes from participants.

### Lack of technical skills and abilities

Students and preceptors identified a lack of technical skills and abilities as one of the characteristics of students with unsafe performance. Students who cannot perform proper initial and continuous assessments, who do not have the skills to perform basic nursing care, who do not have the knowledge of or commitment to safety principles in giving medicine, are likely to have unsafe clinical practices.

One of the educators said:

*Our students do not know the basic principles and skills*,* the principles and techniques are like the foundation of a building*,* the students do not know the basic techniques. They do not know how to take correct blood pressure*,* give safe medication*,* and maintain a sterile field.* (p:2)

Or one of the students said:

*The problem that led to me making a medication mistake and giving multiple doses to the child was that I could not calculate the medication dose correctly. If my educator did not check*,* something bad could have happened.* (p:8)

Or one of the preceptors mentioned:

*Most of the students’ mistakes are about medicines. They usually do not observe the correct method of administration*,* dosage*,* or time.* (p:15)

### Insufficient nontechnical skills and abilities

In addition to their technical skills, students must also have some nontechnical skills to have safe clinical practice. According to the students and preceptors in this study, students with unsafe clinical practice usually cannot prioritize assigned tasks well and complete them on the basis of importance. They do not have sufficient situational knowledge of their abilities and weaknesses, as well as the patient’s emergency situation, and they cannot use proper clinical reasoning to make appropriate clinical decisions by putting together available information. They typically have problems with their professional communication and poor interprofessional cooperation in providing patient care. One of the preceptors says:

*I had students who struggled prioritize their work*,* which often led to mistakes. At the end of a shift*,* there are either unfinished tasks or rush to complete them*,* increasing the risk of errors. For example*,* on one occasion*,* a nursing student who was running late was administered the high-risk drug vancomycin too quickly*,* despite being instructed that it should be infused over more than one hour. This resulted in severe adverse reactions in the patient.* (p:14)

Or one of the students said:

*Once*,* we wanted to get the patient out of bed after the operation so that he could walk. I decided to do this with the help of the patient’s mother*,* and because the nurse was busy*,* we did not ask for her help. The patient almost fell to the ground due to a drop in blood pressure. My decision was not appropriate for that situation at all.* (p:11)

### Limited knowledge

According to the experiences of students, educators, and preceptors, deficiencies in knowledge and cognitive preparation can be among the main characteristics of students with unsafe clinical practice. Students with unsafe clinical practices do not have sufficient knowledge and awareness about procedures, diseases, nursing care, safety principles, preventive safety measures, and job descriptions during internships, which endangers the safety of patients and others. According to their experiences, the knowledge gap can be considered an important factor disrupting safety. One of the students said:

*In general*,* the lack of knowledge and awareness about diseases*,* how to work and the importance of nursing care and equipment can cause us to endanger our patients and increase the possibility of errors.* (p:13)

Another educator said:

*Students are not aware of danger areas*,* or their awareness is very low. They do not know the principles of safety*,* and they are told in a scattered manner. This causes the student to expose himself or herself and the patient to errors. For example*,* the risk of falling among elderly patients increases when bed rails are left open*,* which is a common issue observed in clinical practice.* (p:1)

### Unrefined clinical reasoning and judgment

Students with unsafe clinical performance are perceived as weak on the basis of inadequate clinical thinking and reasoning. These students cannot logically put the available information related to the patient and their problems together and reach a clinical result or decision appropriate to the situation. They cannot properly use the latest scientific evidence, learning and guidelines. Thus, one of the hallmarks of students who may practice unsafely is unrefined reasoning and clinical judgment. One of the educators said:

*Unfortunately*,* we see that the student cannot properly put the available data together in clinical situations and make the appropriate judgment and decision*,* and as a result*,* either he does not intervene at all or he does something wrong and dangerous.* (p:3)

A patient safety control supervisor says:

*Many students cannot apply the knowledge and the theoretical learning that they learned in the class to the clinical practice or use the protocols and guidelines in their work. They only read the theory*,* and this causes errors.* (p:19)

### Nonpatient-centered care

According to the students and preceptors in this study, students with unsafe clinical practices pay less attention to all aspects of care while caring for patients. While performing procedures, they focus only on the procedure and pay less attention to other human, emotional, and even cultural aspects of care. Paying less attention to the preferences and priorities of patients and their families during care is not safe because it may result in students missing important information about client needs, which can result in errors. One of the preceptors says:

*Students only want to do procedures*,* stay in functional things. They only see*,* for example*,* suctioning the patient. They only see the insertion of the patient’s venous line. However*,* who does this to? First*,* one must know if that person*,* does a person have needle phobia. Does he have? His coagulation status? Platelet count? Unfortunately*,* they do not have a holistic view of the patient.* (p:14)

A student said:

*We must be mindful of the religious and cultural differences of our patients. For instance*,* I recall a situation where one of our colleagues was administering a subcutaneous injection of heparin to a woman who was wearing a hijab. When my colleague attempted to locate the injection site by gently touching the patient’s hand*,* the patient immediately protested*,* stating that she did not want to be touched. This led to a tense moment*,* as my friend almost found herself in a disagreement with the patient’s family.* (p:9)

### Inappropriate attitudes about nursing

Students’ attitudes toward the nursing profession can affect their performance. For example, a nonacademic and procedure-oriented view of nursing, a lack of interest, an unenthusiastic attitude toward nursing, excessive self-confidence, a lack of motivation to learn or work, self-centeredness and an unacceptable attitude toward feedback, among other behaviors, were observed in people with unsafe clinical performance. One of the students said:

*According to my own experience*,* the practical and functional dimension of the work that is given to us is much more important from the point of view of many students than diagnosing the heart rhythm and interpreting the patient’s ECG*,* symptoms of diseases*,* and communicating with the patient. If they do the practical work better*,* they will have a greater impact on the educator and the rest of the group.”* (p:8)

Alternatively, one of the educators described the role of interest in the safety of the clinical practice of students:

*I have seen many disinterested students who do not pay attention to the recommendations and create problems for the patient and themselves. They are bored and their number of absences is high*,* and when you ask the reason*,* they say that they are not interested in nursing.* (p:6)

## Category 2: Underdeveloped personal-professional capacity

The participants reported that students with safe clinical practices portray some personal and professional characteristics while attending internships and providing care for patients who lack unsafe practices. A professional-ethical conscience, mental and physical preparation, the type of student behavior during an internship, and the student’s professional appearance can be hallmarks of safe student practices. The absence of these characteristics was considered a hallmark of unsafe practice and was categorized into four subcategories.

### Lack of conscientiousness

Nursing educators and students believe that a lack of moral and professional conscience among students can affect their safe performance. From the participants’ point of view, students with unsafe clinical practices are less likely to adhere to institutions’ regulations and have less moral sensitivity and professional integrity than others are. They do not have enough honesty toward their patients and instructors and show less professional responsibility for their assigned duties. They neglect their educational duties and do not use learning opportunities well. Hiding mistakes and important information is more common among students in unsafe clinical practice. Compared with the other students, they are less disciplined and have a less effective presence during internship hours, and their attention is more focused on virtual social networks and their cell phones. One of the students said:

*Well*,* we do not spend more than a few days in each ward*,* we do not have enough time to learn the rules of each center*,* and the reality is that it does not bother us much. In addition*,* maybe it is not in our learning priorities.* (p:12)

One of the educators said:

*Those who are most unsafe for the patient*,* do not have enough conscience and moral sensitivity and behave in an unprofessional manner. They focus only on performing the procedure and do not see the pain and suffering that the patient is suffering. They are not responsible and even compared to learning*,* they put little effort and are more involved in virtual space.* (p:7)

One of the preceptors says:

*The student creates data because he did not find the sphygmomanometer; he wrote down the blood pressure of the patient himself and made a chart… The unsafe student is easily absent. In addition*,* they give all kinds of excuses to justify their actions.* (p:16)

### Deficit in psychological readiness

Several participants stated that students who had excessive anxiety and haste, an inability to adapt to the conditions of their clinical environments, low self-esteem, and excessive dependence on others most endangered the safety of patients.

One of the preceptors says:

*I had students who could not adapt themselves to the environment and hospital conditions; they have a lot of stress and anxiety; and because of this anxiety; they even do some simple procedures such as a muscle injection incorrectly. You cannot have that anxiety. If he does not control himself*,* he will hurt himself or others.* (p:14)

Another student mentioned the self-deprecation of nursing students, which led to a decrease in the quality of their work:

*The discrimination we see in the departments by the personnel and nurses between nursing and medical students makes us not have a good view of the profession. They talk to them with respect even if they are wrong*,* while they misbehave with us very easily. This induces self-deprecation and lowers self-confidence. This will surely affect our work*,* learning*,* the quality of our work*,* and our seriousness in work.* (p:12)

### Inappropriate professional appearance

Compliance with the uniform issued by the institution and even compliance with personal hygiene and grooming by students can be one of the characteristics of a student with safe clinical practice. Usually, students in safe clinical practice observe personal hygiene, which is uniformly defined by the institution, and to prevent the transmission of infection, they better follow the principles of infection control, such as hand hygiene. In contrast, students who do not follow these guidelines may be unsafe. One of the students said:

*The cleanliness and orderliness of the students are also effective in their acceptance by the patient*,* and in a way*,* it can be a good start or*,* on the contrary*,* a bad start for working with the patient. A student who does not observe his hygiene must also observe the same for the patient. No*,* we saw that such people do not follow the principles of sterile and clean work. For example*,* they do not wash their hands regularly.* (p:11)

### Inappropriate behaviors

The participants described some inappropriate behaviors of students as helpful characteristics in identifying students with unsafe clinical practice. They believed that anger and aggression, inappropriate behavior with the patient and their companions, sleepiness during an internship, and other inappropriate behaviors, such as chewing gum, distraction, and a lack of focus on the patient during the clinical round, are frequently observed among students with unsafe clinical practice.

One of the educators said in this regard:

*Most students who are unsafe*,* unfortunately*,* react badly to the feedback we give them and get angry quickly. They do not accept it and become aggressive.* (p:5)

One of the students said:

*We were changing the patient’s dressing. Our groupmate was standing in front and chewing gum when the professor said to go out and throw it out. Well*,* she does not look good. She was a student who was always reprimanded by various professors for the mistakes she made during her internship.* (p:13)

## Discussion

This qualitative study was intended to identify key hallmarks of nursing students who practice unsafely in clinical practice. The participants described these students as having deficiencies in knowledge, cognitive capacity, and personal professional attributes. Recognizing these hallmarks is crucial for enhancing patient safety during nursing education. Our findings align with prior research by Killam et al. (2013), which characterized safe clinical practice as a multifaceted concept encompassing personal, professional, and functional dimensions [19]. Moreover, our research revealed that students lacking knowledge and cognitive capacity fail to prioritize patients, make sound clinical decisions, and exhibit unprofessional attitudes. Previous studies have emphasized the importance of students acquiring essential skills and cognitive readiness before and during their internships to ensure safe care delivery and successful role transition [1, 35, 36].

Our study also underscores the significance of psychomotor skills and student confidence, echoing previous findings by McPherson et al. (2020), who reported that possessing adequate psychomotor skills, confidence, and mastery in a clinical environment is indicative of safe clinical practice [1]. Similarly, Monique et al. (2019) reported that students with unsafe clinical practice struggled to demonstrate clinical skills and foundational knowledge, often resulting in repeated errors [7]. Brown et al. (2007) noted that such students were ill prepared for internships, unable to perform critical assessments, and delivered suboptimal care [9]. In line with these findings, our research underscores the necessity of technical preparation to ensure safe clinical practice.

Furthermore, our study highlights the importance of nontechnical skills, including task management, situational awareness, teamwork, and interpersonal communication, in ensuring safe student performance. Other studies also report that students with unsafe clinical practice struggle with task management and often perform procedures incompletely [1, 15, 22]. They lack situational awareness and the ability to diagnose and adjust patient care on the basis of clinical cues [16, 37]. Moreover, they face challenges in making appropriate decisions according to patient conditions and encounter difficulties in collaborating with the care team [7, 38, 39]. Effective verbal and nonverbal communication, interaction, and cooperation with healthcare providers and patients are essential for safe practice, and deficiencies in these skills can compromise performance [1, 4, 7, 15, 21, 22]. Failure to report important issues and engaging in false reporting of patient-related matters have also been associated with unsafe clinical practice [9, 15]. To promote safety in clinical learning environments, it is imperative to develop these nontechnical skills in students before their clinical placements. Simulation can be a valuable tool for training these students in healthcare communication, including situations such as disclosing medical errors [40].

Furthermore, our study underscores the importance of cognitive skills for safe practice. Monique et al. (2019) reported that students with unsafe clinical practice lacked basic knowledge and had difficulty demonstrating their knowledge [7]. Knowledge is a fundamental attribute for nursing students to perform safe clinical practice, including understanding body systems, drug functions and side effects, appropriate interventions, and logical reasoning for patient care [16, 17, 19, 23]. The findings of the present study showed that students with unsafe clinical practice cannot properly use the latest scientific evidence, learning, and guidelines, logically combine the available information related to patients and their problems, and reach a clinical result or decision appropriate to the situation. Clinical reasoning is crucial, and students must integrate theoretical knowledge into clinical practice to accurately identify patient problems and select appropriate interventions [6, 37]. Deficiencies in clinical judgment and an inability to recognize and respond to patient conditions were also observed in students with unsafe clinical practice in other studies [13, 21, 37].

Our findings also emphasize the importance of patient-centered care, with unsafe students often focusing solely on procedures and neglecting patients’ holistic needs. Students should involve patients in care planning, considering their preferences and priorities, to provide comprehensive and patient-centered care [41, 42]. In alignment with the present study, previous studies have shown that a lack of patient-centered perspective and a lack of respect and attention to patient needs and patient preferences are unprofessional aspects of students and can be characteristics of students with unsafe clinical practice [1, 17, 19, 38].

Attitude evaluation is one of the most challenging and difficult parts of clinical education and is mostly neglected, leading to the misdiagnosis and correction of unprofessional attitudes being overlooked [7]. In addition to having sufficient skills and knowledge, students in clinical settings are expected to present a professional image of themselves that includes appropriate attitudes and behaviors [4]. The results of this study are in line with those of other studies that have confirmed that warning signs of unsafe practice include disinterest in nursing work, uninterested attitudes toward the nursing profession, overconfidence and doing tasks arbitrarily, selfishness, inattention, lack of motivation to learn or work, and having a defensive attitude toward constructive feedback from educators [1, 16, 21, 23, 43, 44]. Therefore, developing positive and thoughtful attitudes toward care is a fundamental aspect of nursing professionalism and contributes to safe clinical practice.

Furthermore, our study identified underdeveloped personal-professional attributes, such as a lack of conscientiousness, deficits in psychological readiness, behavior during internships, and professional appearance, as hallmarks of unsafe practice. A lack of professional integrity and conscience often leads to noncompliance with standards and unsafe performance [7, 37, 45]. Violating the standards related to fixed nursing practices in recording, reporting, and performing clinical skills can be among the most unsafe clinical practices for nursing students [15]. Ethical awareness and actions based on ethics play a significant role in safe clinical practice [17]. Work ethics and their impact on patient safety have also been discussed in the literature [7, 21, 46]. Accountability is essential for safe clinical procedures, and students must recognize clinical findings, make appropriate decisions, and take responsibility for their actions [6]. Moreover, honesty and accountability are crucial components of safe clinical practice, and students with unsafe clinical practice are characterized by impaired professional accountability for their performance and often refuse to accept responsibility for their actions [8, 16, 23]. Creating an open and honest environment for students to share their experiences and feelings with instructors is important [47].

In this study, clinical educators emphasized the “value of honesty” and agreed that if a student deliberately cheated (e.g., recorded false information in a legal document or falsified the results of a patient assessment), these behaviors are not negotiable and tolerable. In line with the findings of the present study, multiple studies have shown that it is unsafe when students are dishonest through falsifying documents [7, 18, 48].

Additionally, in line with the present study, the results of other studies show that a lack of psychological preparation, such as excessive anxiety [16, 21, 22], haste, difficulty adapting to environmental conditions, excessive self-confidence [18, 23, 45], low self-confidence [16, 45] and dependent personality [49], can be other characteristics of students with unsafe clinical practice. One approach is to have positive interactions with students; also, improving the communication skills of instructors, changing instructors’ and nursing personnel’s behaviors for better acceptance of the students, and supporting them in clinical practice are important to help students adapt to the clinical learning environment [50].

The professional appearance and inappropriate behaviors of students were among the hallmarks of identifying students with unsafe clinical practice in this study. Luhanga et al. (2008) introduced sloppiness as one of the indicators of students with unsafe clinical practice in their study [21]. In the study by Kraja et al. (2021), nursing students acknowledged that adhering to the prescribed uniform increased their level of trust in them and could enhance patient safety [51]. Consistent with this study, previous research has shown that patients attach importance to the appearance of their healthcare providers, considering it a reflection of competence and trustworthiness [52, 53]. Students must know that the first patients’ and visitors’ impressions are determined not only by our tone and expertise but also by their appearance and demeanor. Patients associate professional attire with honesty, knowledge, and high-quality care, which reinforces the importance of professional attire.

Additionally, in line with the present study, the results of other studies show that some unprofessional behaviors, such as anger, aggression, disrespect, verbal or nonverbal insults, inappropriate reactions to the instructor’s feedback, fatigue, and sleepiness, can be other signs of students with unsafe clinical practice [21, 54–57]. Moreover, according to the present study, students may disrupt their interpersonal interactions with patients and affect safe practice by showing unprofessional behaviors such as abnormal eye and face movements, chewing gum, and yawning [4, 7, 21]. More studies may be needed to identify the types and causes of inappropriate student behaviors that can affect safety. After identification, by establishing an open and supportive environment, students can gain the confidence to share their experiences and emotions with instructors, which has been shown to enhance learning and professional development [58–60].

### Strengths and limitations of the study

A strength of this study is the incorporation of different views of clinical instructors, students, and other nursing staff. The cultural, professional, and academic views of faculty members who teach in nursing programs may differ. This study is one of the few qualitative studies in which, in addition to clinical instructors, nursing students, preceptors and other nursing staff involved in nursing education have been used to present their views and experiences regarding students’ unsafe practices. However, this also means that there may be limited depth in the specific context for any one of these groups. Another limitation of the present study may be the concealment of students in presenting their authentic experiences; we tried to solve this problem to some extent by ensuring confidentiality during interviews. Also, despite employing strategies to minimize bias, the researchers acknowledge that their prior relationships with participants may have still subtly influenced the data. However, these relationships also facilitated a level of trust and rapport that may have encouraged participants to share more candid and in-depth accounts of their experiences, potentially enriching the data. This study, like other qualitative studies, has limitations in the generalizability of the results. Limiting this study to one city is appropriate for qualitative research since context may influence the views of the findings; however, that means that it is up to the reader to assess the transferability of these findings to other cultural contexts. Therefore, while the results may be transferable to similar settings, they are not generalizable. Notably, the social and cultural conditions of society may affect the results of the study, so it is necessary to design and implement similar studies in different societies to verify the results.

## Conclusion

The findings of this qualitative study expanded our understanding of the hallmarks of students who may practice in unsafe ways by identifying characteristics that may indicate that a student is in need of more support to practice safely. Early identification and appropriate intervention to guide and manage students with unsafe clinical practices while working in clinical settings are important for patient safety. The findings of the study showed that students with unsafe clinical practice may not have sufficient technical or nontechnical clinical preparation, and they may not have the basic knowledge necessary to work in clinical environments. They may also exhibit personal-professional behaviors that endanger their safety and that of others. Nursing schools must ensure that students have the necessary knowledge, skills, competencies, and personal-professional characteristics to participate in clinical training programs. It is recommended that students with unsafe clinical practices be identified early so that patient safety is maintained and that students are supported in order to correct their weaknesses and improve. The characteristics and unsafe behaviors described in this study can be used by educators to identify early warning signs of poor or unsafe student practices and their management before a critical incident occurs. Continuous support and provision of appropriate training workshops aimed at developing trainers and preceptors by educational institutions can help them identify, guide and manage students with unsafe clinical practice.

## Electronic supplementary material

Below is the link to the electronic supplementary material.

Supplementary Material 1

## Data Availability

The datasets used and/or analyzed during the current study are available from the corresponding author on reasonable request.

## Declarations

### Provenance and peer review

Not commissioned; externally peer reviewed.

## Abbreviations

BSN: Bachelor of Science in Nursing
LPN: Licensed practical nurse
M.Sc.: Master of Science in Nursing
Ph.D.: Doctor of Philosophy
