# Supplementary material for: Hallmarks of nursing students exhibiting unsafe clinical practices: a qualitative study
Source: BMC Nurs. 2025 Apr 18;24:439. doi: 10.1186/s12912-025-03093-x (PMC12007371; doi:10.1186/s12912-025-03093-x)
Supplement: Supplementary file 1 — Supplementary Material 1 [file 12912_2025_3093_MOESM1_ESM.docx]

**Interview Guide**

**Participant Information:**

- **Participant ID NO ....**
- **Age ......**
- **Gender |** Male / Female
- **Participant Semester (For Students) .......**
- **Teaching Experience (For Faculty/Preceptors) ......**
- **Date……**

**Introduction**

- My name is ______________________________, and I am from ______________________.
- This interview is part of a research study aimed at exploring the characteristics of nursing students with safe and unsafe clinical practices.
- The purpose of this interview is to understand your experiences, and it will take approximately __ minutes.
- Your participation is voluntary, and all responses will remain confidential.
- Do you have any questions before we begin?
- May I have your permission to record this interview for analysis purposes?

Consent Obtained: Yes / No

**Interview Sections**

**1. General Experience with Patient Safety**

**(For all participants)**

- Can you share some details about your experience with patient safety issues?
- Have you ever attended a patient safety course or workshop? If yes, please specify.
- Have you ever witnessed or made a mistake in a clinical setting?
- Did you report the error? If so, to whom?
- Do you think the undergraduate nursing curriculum sufficiently covers patient safety topics?

**2. Safe and Unsafe Clinical Practice**

**(For Faculty Members & Clinical Educators)**

- In your experience, what characteristics define a nursing student with safe clinical practice?
- What specific behaviors indicate that a nursing student is engaging in unsafe clinical practice?
- What challenges do you face when identifying and managing students with unsafe clinical performance?
- What institutional support mechanisms are available for handling students with unsafe practices?
- Can you provide examples of situations where a student’s clinical practice posed a risk to patient safety?
- What strategies do you use to guide and correct unsafe behaviors in nursing students?

**(For Preceptors & Clinical Supervisors)**

- How do you assess whether a nursing student is performing safely in a clinical setting?
- What signs do you look for to determine if a student is struggling with clinical safety?
- Can you share a case where a student’s unsafe practice required intervention?
- How do you communicate concerns about a student’s unsafe practice to faculty or hospital administration?

**(For Nursing Students)**

- From your perspective, what does it mean to be a safe nursing student in clinical practice?
- Have you ever felt unsure or unsafe while performing a clinical procedure? If so, what happened?
- What factors do you think contribute to unsafe clinical practices among students?
- Have you received feedback from faculty or preceptors about your clinical safety? How did you respond?
- What support do you think students need to ensure safer clinical practice?

**3. Factors Influencing Unsafe Clinical Practices**

**(For all participants)**

- What institutional, personal, or educational factors contribute to unsafe student practices?
- What role does clinical supervision play in preventing unsafe practices?
- How can nursing education programs improve to better prepare students for safe clinical practice?

**4. Consequences of Unsafe Clinical Practices**

**(For all participants)**

- What are the potential outcomes when a student engages in unsafe clinical practice?
- How does unsafe practice impact patients, fellow students, and healthcare teams?
- What measures should be taken when a student repeatedly demonstrates unsafe practice?

**Closing the Interview**

- Is there anything else you think is important that we have not discussed?
- Thank you for your time and insights. If you have any further thoughts, you can contact us at ____.
